# On-Demand LoRa: Asynchronous TDMA for Energy Efficient and Low Latency Communication in IoT

**DOI:** 10.3390/s18113718

**Published:** 2018-11-01

**Authors:** Rajeev Piyare, Amy L. Murphy, Michele Magno, Luca Benini

**Affiliations:** 1 Fondazione Bruno Kessler, Via Sommarive 18, Povo, 38123 Trento, Italy; murphy@fbk.eu; 2 Department of Information Technology and Electrical Engineering, ETH Zürich, 8092 Zürich, Switzerland; michele.magno@iis.ee.ethz.ch (M.M.); lbenini@iis.ee.ethz.ch (L.B.)

**Keywords:** wake-up radio, wake-up receiver, LoRa, Internet of Things, wireless sensor networks, cyber-physical systems, test-bed and trials

## Abstract

Energy efficiency is crucial in the design of battery-powered end devices, such as smart sensors for the Internet of Things applications. Wireless communication between these distributed smart devices consumes significant energy, and even more when data need to reach several kilometers in distance. Low-power and long-range communication technologies such as LoRaWAN are becoming popular in IoT applications. However, LoRaWAN has drawbacks in terms of (i) data latency; (ii) limited control over the end devices by the gateway; and (iii) high rate of packet collisions in a dense network. To overcome these drawbacks, we present an energy-efficient network architecture and a high-efficiency on-demand time-division multiple access (TDMA) communication protocol for IoT improving both the energy efficiency and the latency of standard LoRa networks. We combine the capabilities of short-range wake-up radios to achieve ultra-low power states and asynchronous communication together with the long-range connectivity of LoRa. The proposed approach still works with the standard LoRa protocol, but improves performance with an on-demand TDMA. Thanks to the proposed network and protocol, we achieve a packet delivery ratio of 100% by eliminating the possibility of packet collisions. The network also achieves a round-trip latency on the order of milliseconds with sensing devices dissipating less than 46 mJ when active and 1.83 μW during periods of inactivity and can last up to three years on a 1200-mAh lithium polymer battery.

## 1. Introduction

The recent Internet of Things (IoT) wave is boosting the development of connected smart low-power devices embedded with various sensors and wireless technologies. The most common IoT devices are sensor nodes that measure physical properties such as vibration, pressure and temperature. For these devices to be “smart”, they are equipped with the on-board processing capabilities to extract useful information from the data before they are sent out to the remote host. These smart devices can make decisions or even take actions by controlling actuators [1]. A common feature of these sensor nodes and, in general, of IoT devices is the capability to transmit data and commands wirelessly.

Most IoT devices are battery powered and expected to have a battery life in terms of years rather than hours. Lifetime is crucial for battery-operated devices, as frequent recharging and replacement will be inconvenient or even impracticable due to the sheer number of IoT devices planned to populate the world [2]. Thus, the design of sensor devices and applications poses several challenges, especially in communication and networking. In fact, the wireless transceiver is one of the most power-hungry subsystems especially when there are many nodes in the network and the required range is on the order of hundreds of meters or even kilometers. Therefore, wireless communication needs to be optimized for improving the energy efficiency and lifetime of IoT devices, as studied in [3].

Today, the most widely-used communication standard for sensor networks in IoT is the IEEE 802.15.4, providing both the physical layer and media access control (MAC) layer specifications [4]. However, one of the main drawbacks of this standard is the limited range of the point-to-point link: up to tens or a few hundreds of meters in the best cases. IoT applications requiring long-range coverage in smart cities and smart environments have pushed the research in wireless technologies enabling novel long-range (LR) communication of several kilometers with power consumption similar to that of standard IEEE 802.15.4 transceivers [5]. The data rate of these long-range chip-sets does of course need to be reduced to achieve greater coverage, but for many low-throughput applications such as environmental sensing, these data rates are adequate.

LoRa is one of these promising low-power long-range technologies, operating in the 868–915-MHz ISM band allowing bit rates between 0.37 kbps and 46.9 kbps and promising ranges up to 20–25 km [6]. LoRaWAN, the MAC protocol for wide area networks, is based on the ALOHA protocol and is ideal for applications with low-traffic and sporadic communication requirements. One of the features making LoRa attractive is its energy efficiency for uplink communication while achieving a long range. In LoRaWAN, the distributed battery-operated sensor nodes send data directly to an always-on gateway. The energy efficiency comes by duty-cycling the main radio transceiver when not transmitting.

### 1.1. Challenges

**Figure 1:**
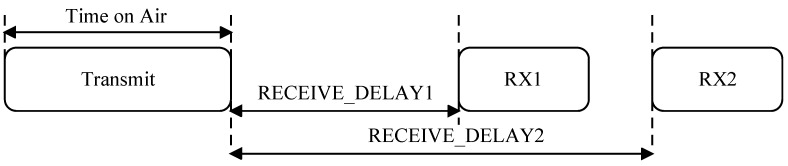
Class A end devices open the two receive windows (RX1 and RX2) after a transmission to receive an ACK or any other downlink traffic from the gateway.

LoRaWAN is mainly designed and optimized for uplinks where the remote end devices disseminate data to the gateway using periodic communication or according to a specified radio duty-cycle. On the other hand, to control the end devices, query data from the specific node or reconfigure node parameters require feedback from the gateway and are absolutely crucial in many Internet of Things application scenarios. For instance, in precision agriculture, hundreds of networked nodes are envisioned to increase production by monitoring the climate conditions and controlling the irrigation and lighting systems remotely. However, in the current LoRaWAN architecture, a fundamental trade-off between downlink traffic, latency and power consumption arises. We rely on Figure 1 to elaborate on this issue. The figure shows the timing diagram of a LoRaWAN Class A end device. The end device first transmits the data to the gateway and then opens up two reception windows (RX1 and RX2), giving opportunities to the gateway for any downlink communication. The transmission slot is scheduled by the end device based on its own uplink requirements. In this communication scheme, the gateway has no control on the end device, and any downlink communication requires waiting or the next scheduled uplink transmission, thereby increasing latency and adversely impacting responsiveness. This affects applications where both low-latency and low-power consumption are required such as structural health [7] and seismic activity monitoring [8]. One may improve the downlink latency by communicating more frequently, but only at the cost of increasing energy at the end devices.

Moreover, LoRa was originally designed to be used as a large-scale star network. As such, this makes it difficult for the nodes to discover other nodes in the network. Without any information regarding neighbors or their transmission schedule, the chance of creating traffic congestion is non-negligible [9]. In fact, even though the amount of data generated by each sensing node can be low, the large number of devices trying to access the wireless channel at the same time can be unmanageable by techniques such as pure ALOHA. These limitations are becoming a serious bottleneck for the success of LoRa-based networks in realizing large-scale deployments, such as environmental monitoring, geolocalization, smart metering and smart grid. Due to the heterogeneous nature of the IoT devices and the huge number of applications, there is a diverse set of requirements that need to be satisfied. These requirements vary in terms of energy efficiency, device lifetime, latency and reliability. Combining different emergent technologies from hardware to software can enable better performance than relying on a single technology.

### 1.2. Approach

Most IoT monitoring applications have data to report which are sparse in time and infrequent such as temperature and humidity with low-throughput requirements. This work targets such applications where the gateway has full control over the network for data collection and node configurations, shifting the communication modality from push to pull based. To facilitate this on-demand communication, we propose a network architecture that adheres to the following requirements:

- Energy efficient: during periods of inactivity, i.e., when there are no data to be transmitted, the end devices must reside in a deep sleep state to maximize device and overall network lifetime.
- Responsive: data must be delivered to the gateway and vice versa in both a timely and reliable manner.

While LoRaWAN targets mainly the first requirement, the latter is not considered at all. To improve energy efficiency and at the same time overcome latency and collision issues due to duty-cycling, in this work, we consider the ultra-low-power wake-up radios (WuR). WuR has the capability to monitor the wireless channel continuously while consuming power orders of magnitude lower than commodity radio hardware typically utilized in wireless sensor platforms [10]. Recent WuR designs also perform address matching with micro-watts of power while waking up the main system only when required [11], avoiding false wake-ups. Due to these features, WuR allows “pure” asynchronous communication by activating the system via interrupts only when a specific signal referred to as a wake-up beacon (WuB) is detected from the wake-up transmitter (WuTX). To benefit from this technology, sensor nodes are equipped with an extra wake-up receiver (WuRX) circuitry and put into low-power modes, waiting for a remote trigger signal. The wake-up receiver, which is typically used in an always-on manner, identifies the incoming wake-up beacon from the sender node. Upon detection of the valid wake-up beacon, it triggers the main node out of sleep mode to exchange data “instantly”, thus reducing latency. Since the wake-up receiver detects signals with very low current, it significantly reduces the wasteful power consumption of idle listening, improving energy efficiency without compromising latency for IoT communications.

In this work, we combine a state-of-the-art wake-up radio with LoRa network technology to fulfill the above requirements. Specifically, we offer the following contributions:

(a) a new network architecture leveraging short- and long-range technologies for enabling low-latency and energy efficient data collection over a two-hop network.
(b) the design and implementation of a new receiver-initiated on-demand TDMA MAC for managing channel access and packet collisions. The proposed MAC offers two modalities for node triggering and allows slot allocation for combating packet collisions. On-demand TDMA achieves an improvement of at least 1.72× in terms of latency and an extended node lifetime of 1.4× with 100% system reliability over the traditional channel sensing scheme for LoRa. This new feature still works with the standard LoRa, but improves overall performance with an on-demand TDMA scheme.
(c) introduce an analytical model to quantify the data collection latency for on-demand TDMA in broadcast and unicast mode.
(d) the performance evaluation of the proposed network architecture and MAC using an indoor testbed composed of 11 sensor nodes.

The paper presents the relevant background and related works in LPWANs and wake-up radios in Section 2 and Section 3 respectively, then outlines the proposed network architecture and MAC design in Section 4 and Section 5 offers the experimental setup followed by an evaluation and the results of the full system design. Finally, Section 6 provides concluding remarks and possible future directions.

## 2. Background

We first give the required background on the wireless technologies and techniques that we exploit in this work to achieve energy-efficient sensing and data collection. We begin with background on LoRa followed by ultra-low-power wake-up radios.

### 2.1. Low-Power Long-Range Communication: LoRa

To extend the IoT device connectivity, recently, a new set of wireless technologies appeared that can attain long-range communication, up to several kilometers, with power demands similar to those of ZigBee devices [12]. These technologies are grouped under the label of low-power wide area networks (LPWANs) and operate at sub-GHz frequencies. To achieve a long range, three main modulation techniques can be used: ultra-narrow band, spread spectrum or narrow band.

The key technology players that are trying to fill the gap in the long-range device-to-device (D2D) communication are SigFox, LoRa, NB-IoT and weightless-N [13]. LoRa has been very successful in the market due its open protocol standard and chirp spread spectrum (CSS) modulation technique that allows recovering data from weak signals even below the noise floor. Due to this technique, communication is more resilient to interference and is inherently secure. To control the trade-off between the transmission range and data rate, LoRa allows configuring the radios with different spreading factors (SF) within the range [6,12]. A higher SF increases receiver sensitivity and thus range, but decreases the symbol rate. In this work, we use three different SF settings: SF7, SF9 and SF12, which offer data rates of 21.87 kbps, 7.03 kbps and 0.976 kbps, respectively. Further, communications with different SFs do not interfere with one another and create a set of “virtual” channels, increasing the capacity of the gateway.

**Figure 2:**
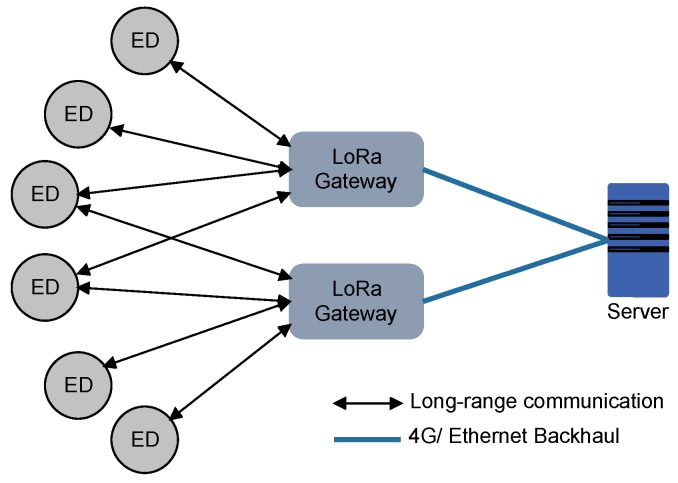
LoRa network topology.

The LoRa physical layer can be used with any MAC layer, and currently, LoRaWAN is the only standardized MAC. In the LoRaWAN architecture [14], the network is formed by one or more gateways and multiple end devices organized in a star-of-stars topology, as illustrated in Figure 2. The end-device changes the channel in a pseudo-random fashion for every transmission and communicates directly to the gateway, which then forwards the data to the server. LoRaWAN defines three classes of end devices (A, B and C). For Class A, each uplink transmission from the end device is followed by two downlink windows, as illustrated in Figure 1. Class A is the most power efficient, as nodes only wake up to send data. Class B devices provide the Class A functionality plus open extra receive window at scheduled times. Class C devices are always listening to the channel, except when they are transmitting.

### 2.2. Wake-Up Radios

Wake-up radios are a recent hardware technology to improve the energy efficiency in communication. To achieve ultra-low-power consumption, the wake-up radio works with on-off keying (OOK)-modulated signals. This drastically simplifies the wake-up receiver circuitry where only a few discrete components are sufficient to construct OOK signal detection circuitry [11,15]. WuRs are always-on devices that need to operate at a very low power. As a consequence, they usually have a lower sensitivity and bit rate than standard transceivers used in sensor networks. To extend the range of the wake-up radio communication, most designs operate in the sub-GHz band [11,15,16].

In this work, we employ a state-of-the-art wake-up receiver that consumes only 1.80 μW in continuous listening mode [11]. This wake-up receiver has been tuned for the 868-MHz band and has a receiver sensitivity of −50 dBm, reaching up to 20 m line-of-sight indoors. To trigger the wake-up receiver, we have exploited the OOK modulation feature of the Semtech SX1276 LoRa module to cross communicate between a LoRa transceiver acting as a wake-up transmitter and a wake-up receiver, both operating in the 868-MHz frequency band. In addition, this wake-up receiver design also provides address decoding capabilities at the cost of a few additional micro-watts, by integrating an ultra-low power microcontroller unit. In this work, we rely on the addressing feature of the wake-up receiver to prevent erroneous activation.

## 3. Related Work

Various IoT applications and wireless protocols use a many-to-one communication paradigm where sensing nodes transmit information to a single node or gateway. We next present some of the existing approaches for many-to-one communication in long-range networks followed by wake-up radios.

### 3.1. Long-Range Communication Schemes

Carrier sense multiple access with collision avoidance (CSMA/CA) is one of the most widely-used MAC protocols in wireless local area and short-range networks for efficient many-to-one data collection. However, as the number of devices in LPWAN increases, the carrier sensing mechanism becomes less effective at reliably detecting channel activity, negatively affecting network performance [17]. Therefore, LPWAN technologies such as LoRaWAN and SigFox have adopted pure ALOHA access protocols for uplink communication [18].

ALOHA is an asynchronous protocol where the end devices communicate when they have data ready to send, either scheduled or event-driven. Several studies have evaluated the scalability of ALOHA using simulations by taking into account the physical and link layer information in terms of latency, reliability and throughput [9,18,19,20]. Most of these studies conclude that although ALOHA operates well under low-traffic loads, and it suffers from uplink traffic congestion as the number of network devices increases due to its inability to check whether the channel is busy before transmitting. Pop et al. [21] also studied the effect of bidirectional communication and its impact on the throughput of LoRa networks. The findings indicate that use of downlink traffic, e.g., data and acknowledgments, can corrupt the uplink packets reducing the overall throughput and network reliability. Therefore, for networks that do not require high reliability, use of acknowledgments and retransmission attempts should be chosen carefully.

Recently, an effort has been made in [22] to increase the scalability for LoRa networks by dividing the available bandwidth into single synchronous downlink channel and several asynchronous uplink/downlink channels. The gateway sends a beacon to synchronize the nodes that also carries the information specifying the allowed transmission power and spreading factors to be used by the nodes for uplink transmission. Nodes upon wake-up listen to the latest beacon to synchronize the information and then transmit the data in an ALOHA manner. Although the proposed RS-LoRa MAC in [22] mitigates packet collisions by nearly 20% compared to standard LoRaWAN, it does not fully eliminate them as the uplink messages could still collide with beacons from neighboring gateways.

Due to these issues, the ALOHA MAC can become a bottleneck for the success and scalability of LoRa networks as deterministic operation cannot be guaranteed. Motivated by the above state-of-the-art, we propose an on-demand TDMA that provides a deterministic performance in terms of network latency w.r.t. to the network size, which is highly desirable for low-power low-latency applications. Moreover, most of the above analyses are restricted to simulation and consider only homogeneous networks, thus limiting the variety of applications to which LoRa can be applied; for instance, applications that require both short- and long-range capabilities [23] to provide different services. To open up new application scenarios in this direction, we exploit the feasibility of combining heterogeneous networks. Furthermore, instead of taking a simulation approach, we evaluate the full network performance via testbed analysis. To the best of our knowledge, on-demand TDMA is one of the first to test receiver-initiated MACs in a wake-up radio setting with a LoRa-WuR testbed.

To improve bandwidth utilization, some studies have proposed a listen before talk (LBT) procedure before transmission [24,25] for Class A devices. The LBT mechanism allows multiple users to share the same channel by continuously monitoring the channel so as to transmit only when it is free. LoRa transceivers provide a special mode called channel activity detection (CAD) for channel occupancy by detecting a preamble. In case a preamble is detected, the transmitter backs off for a random period of time, then performs channel sensing again. Although LBT allows a high degree of collision avoidance, it increases the power overhead required by the transmitting nodes for channel sensing, a factor crucial for battery-powered sensor nodes. Moreover, LBT also contributes to packet delays due to the exponential back-off mechanism. On-demand TDMA on the other hand efficiently triggers the nodes for data collection over wake-up radio while packets are tightly packed in the available slots without additional delays. As a result, on-demand TDMA achieves high system reliability without compromising energy efficiency and latency.

### 3.2. Wake-Up Radio-Enabled MAC

To extend the lifespan of batteries, most IoT devices resort to duty cycling mechanisms, where the device periodically wakes up from the sleep mode to retrieve new information. Although this allows saving power, it also introduces limitations. First, the device periodically wakes up even if there are no data to exchange, thus wasting energy. This behavior is known as idle listening. Second, using long sleep intervals increases data latency [26].

To address the above issues, a novel hardware-based technology called wake-up radio (WuR) has gained popularity in the WSN community. A recent survey [10] highlights that major work involving WuR has been on improving hardware design to achieve better communication characteristics with low-energy demand. To reap the benefits of this new technology, a few studies have started to integrate low-power protocols for remote triggering and on-demand communication. Sparse Topology and Energy Management (STEM) [27], based on TI-MAC, uses a dual-radio approach to differentiate data and wake-up channel while relying on the regular high-power radio as a WuR to achieve coverage up to 20 m. Yang et al. [28] propose a pipelined tone wakeup scheme for data and tone detection using separate radio channels. In PTW, the nodes transmit a broadcast tone before sending data. As the wakeup procedure is broadcast, all nodes within range are triggered. From the point of view of application scenarios, PTW enables fast network-wide wake-up; however, it is less energy efficient, as all nodes, upon wake-up, try to transmit concurrently, causing collisions and energy overhead. In this work, we also take advantage of the broadcast scheme for network-wide wake-up with reduced delay; however, to overcome the issue of packet collisions, we allow each node to transmit at different times using an on-demand TDMA scheme.

A few WuR receiver-initiated (RI) protocols shift this burden to the receiver [29,30]. In RI systems, the task of communication initiation falls to the receiver node, often the sink or the base station, broadcasting a beacon to either request data or to announce its readiness to receive data. The benefits of adopting a receiver-initiated scheme are the following: (i) no collisions, as the receiver is in charge of pulling the data from the individual sensing nodes when required; (ii) asynchronous communication, due to the fact that RI protocols do not require tight network-wide synchronization, so a pure asynchronous communication can be achieved.

The on-demand TDMA scheme developed in this work adopts a receiver-initiated asynchronous MAC via wake-up radios for time synchronization and data collection, reaping the benefits mentioned above. This allows the proposed MAC to be in full control of when to retrieve data from the nodes without congesting the network while achieving 100% reliability.

Other works have also proposed multi-hop data dissemination and collection using WuRs. Wake-up radio MAC (W-MAC) [31] provides bi-directional functionality to support multi-hop communication and can be used for small or large networks. In addition, W-MAC supports addressable and broadcast signaling for reducing false wake-ups, a feature also supported by on-demand TDMA proposed in this work. On the other hand, ZIPPY [32] and BLITZ [33] both depend on synchronous transmissions over wake-up receivers for network flooding and data dissemination. In contrast, on-demand TDMA achieves network synchronization in a fully-asynchronous manner on the order of tens of microseconds, i.e., 95 μs, as opposed to ZIPPY and BLITZ, which rely on frequent synchronous transmissions.

While various WuR MAC protocols have been designed and tested, recently, an attempt has been made to fuse the short-range WuR with a long-range transceiver reaching up to several kilometers to extend the connectivity of devices. The authors in [34] have presented a hardware design that uses dual radios to reduce power consumption for LoRa devices. Through analytical analysis, the study indicates that using the wake-up receiver can substantially decrease uplink/downlink latency for LoRa networks and increase energy efficiency. Although the work in [34] has been the starting point in this direction, it does not support data collection from multiple nodes within the star network and has not been experimentally evaluated in a testbed with more than two nodes. In contrast, the current work presents an on-demand MAC protocol that leverages a WuR and a LoRa transceiver with the goal of reliably collecting data from multiple sensor nodes without data collisions, yet maintaining high reliability and deterministic operation. We also go beyond prior works and carry out an evaluation via testbed analysis with multiple sensor nodes. Moreover, we also carry out a systematic evaluation with different LoRa radio settings to explore the benefits and the trade-offs it has on energy and overall data latency.

## 4. Energy-Efficient Data Communication Network and Protocol

Next, we outline our proposed network architecture and our on-demand TDMA protocol for LoRa communication to achieve the goal of improving energy efficiency without compromising data latency and network reliability. The network architecture is designed to realize many-to-one and one-to-many communication.

### 4.1. Network Architecture

**Figure 3:**
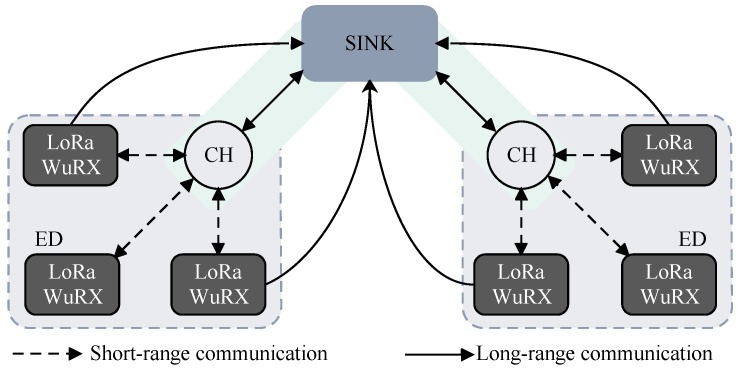
Heterogeneous IoT topology for sensor and actuator networks.

In this work, we propose a heterogeneous IoT network where the gateway is in charge of collecting data from a specific end device or cluster of devices when required. To maintain the low-power state, the end devices offer a dual-radio interface: a wake-up receiver for short range radio (meters) and a LoRa transceiver for long-range communication (kilometers). With pure on-demand asynchronous communication over the wake-up receiver, the end devices need not periodically or continuously listen to the channel, thus overcoming the issue of idle listening and latency with improved energy efficiency. Unlike LoRaWAN, where a gateway communicates directly to the nodes, in the proposed network architecture, we partition the network into clusters. The clusters are comprised of sensor and actuator nodes that form a star topology, and any down-link communication from the gateway to the nodes (one-to-many) must go through the cluster heads due to the short communication range of wake-up receivers, as illustrated in Figure 3.

In detail, the network is composed of three different nodes:

1. The end device (ED) is responsible for sensing and equipped with both a wake-up receiver and a LoRa transceiver. The EDs are battery powered and, therefore, will spend most of the time in a low-power mode, i.e., deep sleep state.
2. The cluster head (CH) is in charge of managing the nodes in the cluster and relaying commands from the gateway to the EDs. Depending on the application scenario and the energy availability, the CH can either operate in a duty cycle mode or can always be listening. The nodes designated as CHs are also equipped with both radios. Each CH is assigned a unique ID address allowing the sink to query each CH at a time, thus reducing the interference from other clusters.
3. The sink acts as a gateway and is assumed to have no energy constraints and will be wall-powered. Therefore, the sink can be always on and listening for any incoming data. Unlike ED and CH, the sink only offers long-range communication without the wake-up radio interface.

The communication directionality can differ depending on the node classification. The transmission between the sink and the CH is bidirectional (sink⟷CH), allowing the gateway to update network parameters or collect data from the cluster heads and end devices directly. The CH and the EDs also communicate bidirectionally (ED⟷CH), so that the CH can trigger the EDs and the EDs can query neighboring nodes without passing through the gateway every time. Moreover, in urban scenarios, the presence of obstacles such as buildings or foliage could deteriorate the link quality from the ED to the sink [35]. Under this condition, the EDs could relay the messages to their CH, which is expected to have good link conditions, which could then forward the message to the sink. On the other hand, EDs communicate unidirectionally to the sink (ED⟶sink), maintaining a single-hop communication scheme as in the LoRaWAN architecture.

To improve the energy efficiency of the EDs, we propose an on-demand TDMA scheme (Section 4.2). This on-demand data request scheme interrupts the EDs through remote triggering using the wake-up beacon. In this scenario, the sink, which can be located a few kilometers away from the clusters, initiates the data collection by first sending the data request to the CH using the LoRa physical layer. The CH then disseminates this to the EDs by transmitting a wake-up beacon, thus exploiting the short-range feature of the wake-up receivers. Since each ED is equipped with a wake-up receiver, either one or all EDs can be triggered by the CH through an addressed or broadcast-based wake-up beacon. Finally, the EDs respond to the request by offloading data directly to the sink (many-to-one), similar to nodes in the LoRaWAN architecture. The end devices then return to the deep sleep listening state until the next wake-up event is detected.

The proposed architecture together with on-demand TDMA leads to interesting performance gains for uplink/downlink communication in terms of energy and latency in contrast to the LoRaWAN architecture. Next, we present this on-demand TDMA scheme and its inner workings.

### 4.2. On-Demand TDMA MAC Design

One of the main contributions of this paper is a novel on-demand TDMA: a low-complexity, reliable and energy-efficient data collection scheme that leverages dual radios for the LoRa network in IoT applications. In all cases, our goal is to pull data from all nodes in the most energy-efficient and low-latency manner. Therefore, the core of the proposed MAC is receiver-initiated on-demand communication providing network-wide data collection with reduced packet collisions.

We developed two mechanisms to achieve the above goal, explicitly the two primitives of the wake-up radio, namely unicast and broadcast. Unicast is useful for applications that need to gather data only from specific sensor nodes in the network. For instance, the base station triggering a particular sensor located in a house or on a farm for the latest measurements. As an alternative, the trigger can be broadcast with the intent to activate all sensors in range for gathering data such as the status of all fire alarms on the same floor. Next, we discuss these two different modalities offered by our proposed MAC.

#### 4.2.1. Unicast TDMA

In unicast mode, we exploit the addressing feature of the wake-up radio to trigger and pull data from a specific ED. This mode can further be extended to pull data from other EDs or nodes in the cluster by sending multiple unicast requests. Initially, the sink commences the data request by transmitting the command beacon to the CH using its long-range transceiver. The sink then switches to receive mode and samples the channel for any incoming data packet. Since the transceiver at the CH is always on and listening, it receives this request “almost immediately” and then proceeds to transmit the addressed wake-up beacon to the specific sensor node as requested by the sink. As introduced in Section 2.2, the always-on wake-up receiver on the ED detects this beacon, decodes the embedded address and transitions the main MCU out of deep sleep. The ED then transmits the data packet to the sink using its long-range SX1276 transceiver. After the reception, the sink either repeats the request process with other EDs using the unicast procedure or can fall back to continuous listening mode. The benefit of this modality is that the unicast TDMA not only eliminates the idle listening at the sender (ED) and the receiver (sink), but also reduces the downlink data latency (sink⟶ED). This reduction is achieved as the sink does not have to wait until the next uplink transmission to acquire fresh data as opposed to LoRaWAN specifications.

#### 4.2.2. Broadcast TDMA

In contrast to unicast mode, here, we leverage the broadcast feature of the wake-up radio to trigger and pull data from all the nodes in the cluster using a single broadcast beacon reliably and efficiently. The goal is two-fold. First, the broadcast beacon allows waking up all the nodes in range for data collection reducing latency w.r.t. to the unicast mode where several addressed wake-up beacons are transmitted for activating each different node. Second, transmitting a single beacon saves energy both at the sink and the CH side as multiple beacon transmissions are expensive. We will quantify these savings when presenting our findings under the results in Section 5.3.3. However, the former introduces collisions, as all the nodes may try to transmit data concurrently upon successful wake-up, causing network congestion. To mitigate this, we have implemented a simple, yet robust slot scheduler atop RI-MAC, thus the name on-demand TDMA. The slot assignment per node allows full utilization of the bandwidth while reducing the channel activity and eliminating packet collisions in the network. We show experimentally in Section 5.3.1 that a system reliability of 100% is achievable using the proposed on-demand TDMA technique.

**Figure 4:**
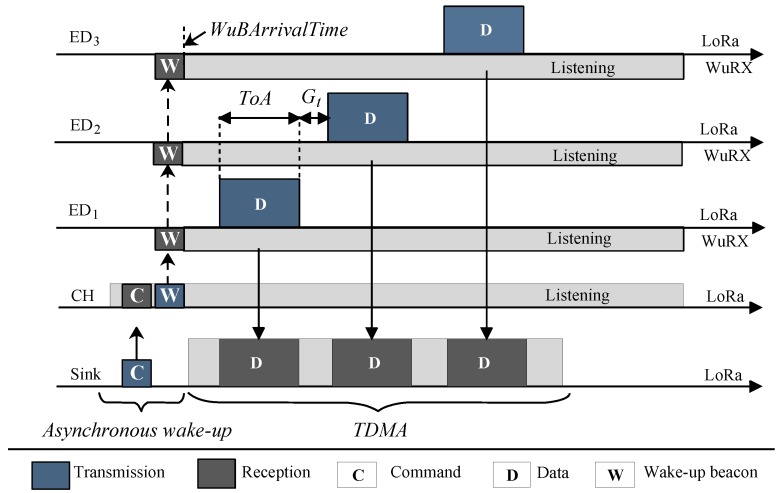
Receiver-initiated on-demand TDMA MAC overview.

Figure 4 exemplifies the operation of on-demand TDMA using short-range and long-range radios. Similar to the unicast case, the sink initiates communication via the CH. This time, the CH transmits a wake-up beacon with a network broadcast address for activating all the EDs within the cluster. After sending the trigger command, the sink opens a receive window within which it accepts all the incoming data from the EDs via its LoRa transceiver.

**Figure 5:**
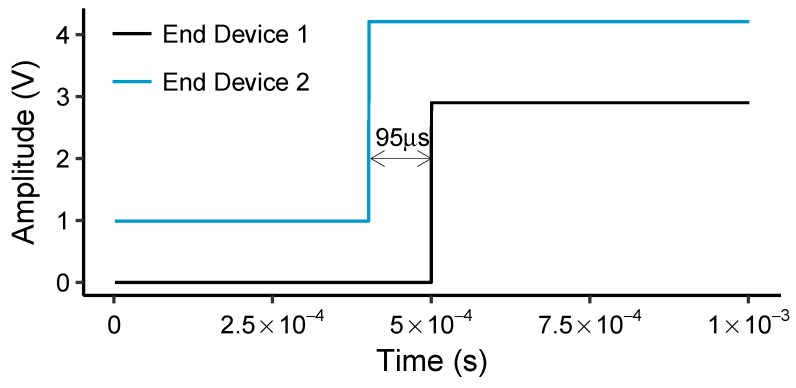
Time synchronization measurement between two end devices over the wake-up receiver.

For proper operation of the on-demand TDMA, all nodes in the cluster need to agree on the time slots; therefore, clock synchronization is required. We leverage the wake-up beacon to provide a fine-grained time synchronization among the EDs achieved by the asynchronous network wake-up, marking the start of an epoch. To measure this synchronization accuracy, we programmed the two end devices to assert their specific GPIO pin after receiving the correct wake-up beacon. Figure 5 illustrates the times at which these end devices raise their GPIO pins. The difference is measured as 95 μs, indicating that clock synchronization on the order of tens of microseconds is achievable using wake-up radios without requiring complex time synchronization algorithms.

#### 4.2.3. Slot Allocation

All the EDs are now active and synchronized. First, each ED enters into a slot reservation phase where the EDs occupy the slot according to their node ID, $N_{id}$ i.e., the node with ID 1 occupies the first slot, while the node with the maximum ID occupies the last slot. In the current implementation, node IDs are statically assigned a priori at network configuration time. The ED measures the exact time when it was triggered by the CH, denoted as WuBArrivalTime. Each node then computes the start of its time slot from the WuBArrivalTime as:

(1) $$T_{NextSlot}=WuBArrivalTime+\left(ToA_{pkt}+G_{t}\right)N_{id}$$

The slot size is determined by computing the time-on-air, $ToA_{pkt}$, using Equation (A1) (See Appendix A) for the LoRa data packet depending on the payload size with a pre-defined guard time, $G_{t}$, of 6 ms. The guard time guarantees that the window is large enough for the transmission and compensates for clock drift, which may be detrimental with an increasing number of EDs. Finally, the EDs start transmitting the data packets over the LoRa module according to the slot schedule, as illustrated in Equation (1).

Moreover, to abate extra delay and transmission consumption, no acknowledgment frames are exchanged between the sender and the receiver. For systems that require 100% reliability at the expense of latency, an acknowledgment can be easily incorporated into our protocol to retransmit in case of packet loss. The above two proposed modalities are evaluated next through experiments, and the results are presented in Section 5.3.

## 5. Experimental Evaluation

This section presents the in-field results of the on-demand TDMA protocol evaluated through a test-bed analysis. Next, we describe the details of the experimental setup, evaluation metrics and the results.

### 5.1. Experimental Setup

#### 5.1.1. Testbed

**Figure 6:**
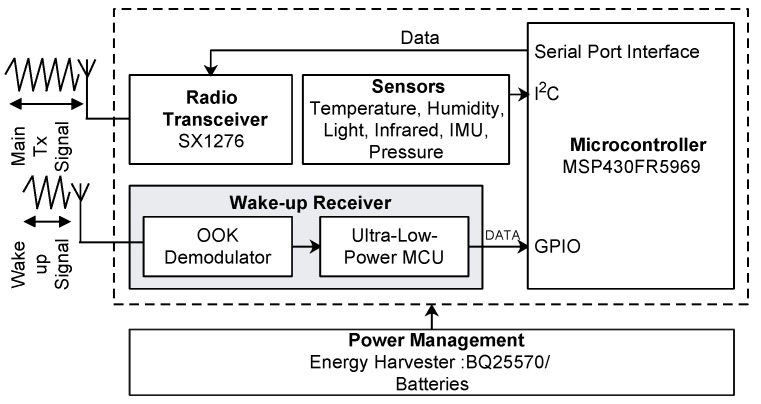
Hardware architecture of the heterogeneous communication mote used for testbed evaluation.

To show the proof of concept, we deployed a total of 11 wireless sensor nodes in an indoor office environment based on the prototypes previously developed by some of the authors of this work [36]. Moreover, since the wake-up receiver hardware is custom designed to work with the LoRa module, a small-scale testbed was the most feasible solution. Of the 11 nodes, 9 are designated as end devices (EDs) responsible for sensing, while one acts as the cluster head and one as a sink. The hardware architecture of the wireless multi-sensor dual-radio platform is shown in Figure 6 and includes the following main components: microcontroller, radio transceiver, wake-up receiver, sensors and the power management unit. The platform integrates a multi-purpose SX1276 wireless transceiver from Semtech Corporation that supports different modulations such as (G)FSKand OOK, as well as the LoRa physical layer. To use it as a wake-up transmitter, the SX1276 is configured for transmission using OOK modulation where the information is sent using ‘1’s and ‘0’s. An OOK 1 sub-bit is produced by transmitting a large amplitude carrier, while an OOK 0 sub-bit is produced by sending nothing, i.e., the transmitter is turned off.

Together with the SX1276 transceiver, each end device is also equipped with a wake-up receiver coupled to an 8-bit ultra-low power PIC12LF1552 MCU for selective triggering. The wake-up receiver consumes 1.80 μW in listening mode and 284 μW when it is actively receiving and decoding the preamble or address. The maximum bit rate supported by the wake-up receiver is 1 kbps. Finally, the platform uses two separate antennas, one for the main transceiver and the other for the wake-up receiver. Table 1 depicts the power consumption of the various modes of the platform measured in-lab with a N6705B DC Power Analyzer.

**Figure 7:**
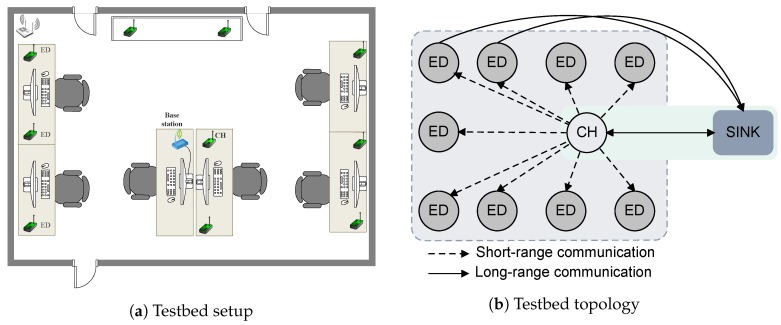
Network topology and indoor testbed deployment.

Although, all the nodes are equipped with the LoRa and the wake-up receiver, in our evaluations, only the EDs use both radio interfaces. The sink node acting as the base station is connected to the laptop to log data and to verify transmissions. Figure 7a captures the testbed deployment, while Figure 7b illustrates the topology used in the experiments with different node designations.

#### 5.1.2. Radio Settings

In LoRa networks, switching to a different data rate affects the receiver sensitivity. At high spreading factors, i.e., low data rate, LoRa packets can be received at a much longer range with high reliability. To explore this, we selected three different LoRa radio configurations as summarized in Table 2. With Setting 1 (SET 1), we chose the most robust transceiver setting with the lowest data rate (0.976 kbps) and highest spreading factor, leading to the transmission time of 264 ms. Setting 2 (SET 2) was chosen to represent a mid-range data rate of 7.03 kbps with time-on-air of 31 ms, while Setting 3 (SET 3) represents the shortest airtime of 9 ms with bit rate of 21.87 kbps.

All experiments were performed with an 8-byte application payload, a 2-byte wake-up address and the guard time $G_{t}$ = 6 ms. The value of $G_{t}$ was chosen such that more data slots can be fitted per epoch without overlapping the slots. The transmission power for LoRa and the OOK signal was fixed at +14 dBm. In contrast to LoRaWAN, in all our experiments, the LoRa radios were configured to use a single channel for communication. Therefore, to provide a fair comparison, we have evaluated the performance of the on-demand TDMA against the LBT protocol with random backoff. The random backoff interval for LBT is restricted to be between {0, 2} s.

#### 5.1.3. Ambient Noise Floor

**Figure 8:**
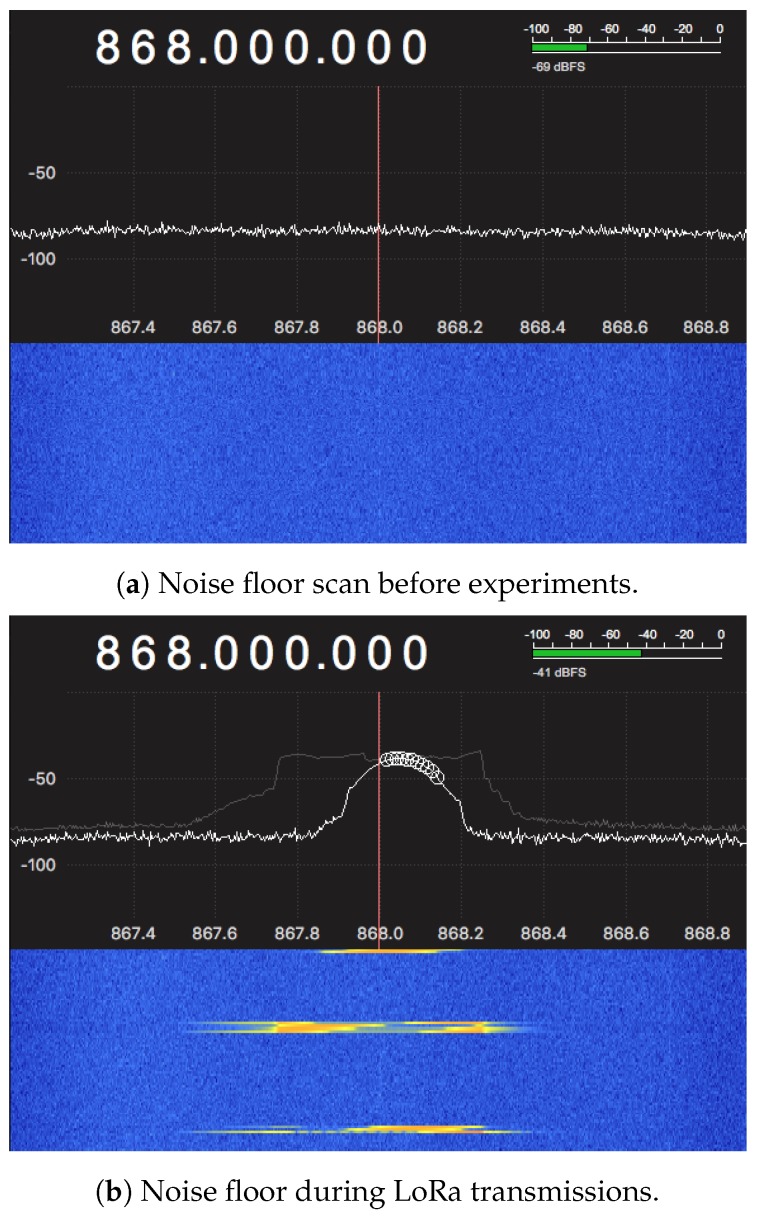
Measurement of the noise floor in the office testbed environment.

Before starting an experiment, the noise floor of the ambient environment was measured using the NooElec software-defined radio to check if there were any nearby devices operating in the 868-MHz band. This can be detrimental as both the radios, LoRa and the wake-up receiver used in this work are operating in this frequency band, and any noise on the channel can cause false wake-ups or interference. Figure 8 shows the noise activity for the measured band together with the waterfall plots captured from the SDR software. The plot in Figure 8a indicates no interference or devices operating nearby in this frequency. As a comparison, Figure 8b shows the noise floor during LoRa transmissions with a peak near the center frequency.

### 5.2. Evaluation Metrics

To evaluate the performance of the proposed protocol, we defined three metrics: (i) packet delivery ratio (PDR), representing how reliable the protocol is, computed end-to-end from the sink to the sensing node. To detect the missing packets, each payload is sent with an increasing sequence number. If the sequence number is seen at the sink, the data packet is counted as received. (ii) Round-trip time latency (RTT) is measured as the time difference from the initial trigger by the sink and the time to receive the data from all the sensing nodes. For instance, in an application such as alarm generation, reducing end-to-end latency is a key objective. (iii) The radio duty-cycle (RDC) is computed as the ratio of time where the radio is active and the interval between two successive wake-ups: $RDC=\left(t_{TX}+t_{RX}\right)/\left(T\right)$, where $t_{TX}$ and $t_{RX}$ are the time when the radio is on either transmitting or receiving/listening and *T* is the total length of the period, expressed in milliseconds. This is a platform-independent metric indicating how energy efficient the protocol is; (iv) Energy consumption is computed as the total energy consumed by the network nodes including the consumption of the wake-up receiver to collect all the pieces of data.

All metrics have been computed based on 500 packets exchanged between the nodes using the static configuration as shown in Table 2 over three different trials. We also show the preliminary results of the scalability test and its impact on latency and power in the next section.

### 5.3. Network Performance Analysis

Next, we present the results obtained from the testbed experiments. Here, we concretely demonstrate the performance in terms of network reliability, latency and energy efficiency of the on-demand TDMA when integrated into the proposed network architecture. For all the experiments, we vary the network load by exploring a range of inter-packet intervals (IPI) from 10–60 s. For all the evaluation metrics, we first compare the broadcast TDMA against listen before talk, followed by Unicast TDMA mode.

#### 5.3.1. Packet Delivery Ratio

**Figure 9:**
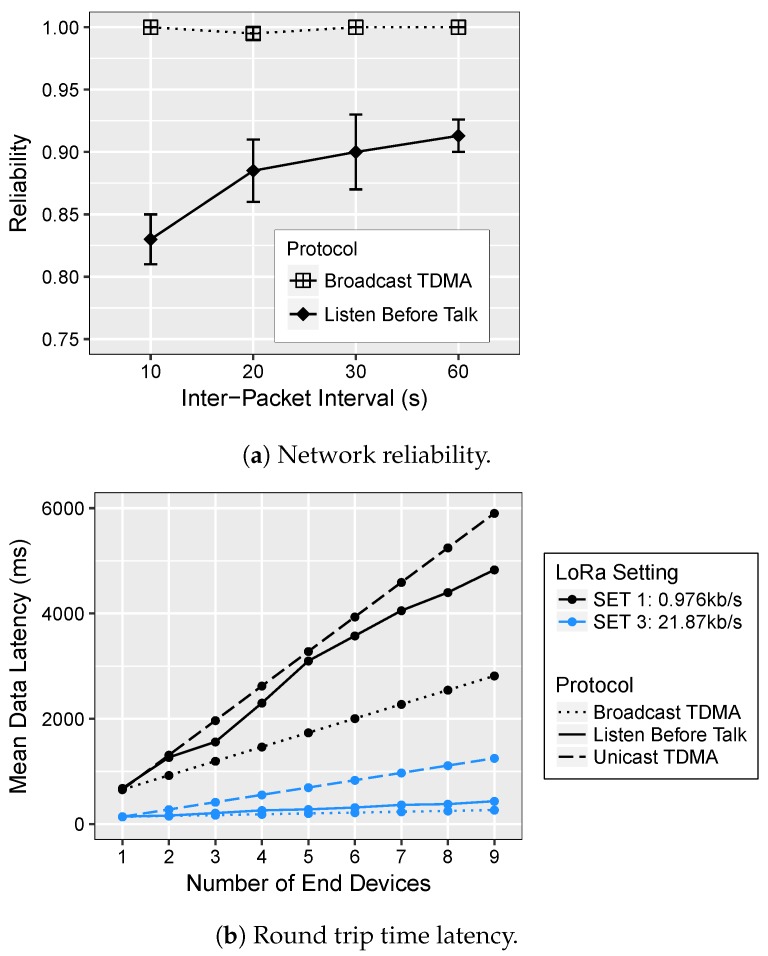
Network reliability and latency evaluation using different LoRa radio setting and protocols.

We begin our evaluation by considering the data collection reliability of the network, shown in Figure 9a, in terms of generated data packets and those successfully received by the sink. The vertical bars indicate the maximum and minimum PDR achieved in each trial. We first observe the PDR of 100% for broadcast TDMA for varying IPIs. The higher PDR is attributed to the facts that: (i) each ED transmits in its own allocated slot, reflecting the ability to handle higher bandwidth without collisions; (ii) the distance between the nodes in our experiments is small; therefore, all the trigger samples were correctly received by the wake-up receivers.

On the other hand, the average PDR for the LBT protocol varies between 83% and 91% for varying traffic. This is because the CAD feature is only useful to detect LoRa preamble symbols. As CAD is not able to detect all transmissions, especially when the preamble has been already sent, this causes packet collisions at the receiver, dropping some packets, resulting in lower reliability. Thus, for the same IPI, different LoRa radio settings could affect the PDR. If the LoRa setting has a long preamble and short data packet, then CAD would be more effective.

#### 5.3.2. Latency

Next, we turn our attention to the network latency as shown in Figure 9b. In this set of experiments, all the nodes in the network used the same configuration $Setting=\left\{SF,BW,CR,CF,TP\right\}$. We compared the three different LoRa radio configurations and their impact on the overall network latency using both the protocols, on-demand TDMA and LBT, and two modalities of on-demand TDMA. Since the on-demand TDMA offers a level of determinism that is not attainable with pure ALOHA or LBT MAC protocols, the data latency can be modeled. For latency, the round-trip time is computed as the difference between the sink node transmitting the command packet and receiving all the data from the end devices. These delays include the overhead of 17 ms required by the wake-up receiver to receive a 2-byte wake-up address at the data rate of 1 kbps, decode the packet and generate a trigger to the main microcontroller. For the unicast mode, the latency, $L_{Ucast}$, is given by Equation (2), while for the broadcast mode, $L_{Bcast}$ with slot assignment is expressed in Equation (3), where $t_{\left(S\to CH\right)}$, $t_{\left(CH\to ED\right)}$, and $t_{\left(ED\to S\right)}$ is the time required to send the command from the sink to the CH, relaying the trigger from the CH to the ED and transmitting the data packet to the sink, respectively, according to the number of EDs, $N_{nodes}$, in the cluster.

(2) $$L_{Ucast}=\left[t_{\left(S\to CH\right)}+t_{\left(CH\to ED\right)}+t_{\left(ED\to S\right)}\right]N_{nodes}$$

(3) $$L_{Bcast}=t_{\left(S\to CH\right)}+t_{\left(CH\to ED\right)}+\left[ToA_{pkt}+G_{t}\right]N_{nodes}$$

##### Broadcast TDMA vs. LBT

For both protocols, the latency increases linearly with the increasing number of EDs for each setting as captured in Figure 9b. For higher numbers of nodes, however, on-demand TDMA compares favorably to LBT because nodes are able to send packets without collisions in their own slots. The only extra delay that occurs per transmission is the 6-ms guard time used to compensate for clock drifts. On the other hand, nodes using LBT must compete for the medium and backoff whenever the other node is transmitting. The competition for medium is low with fewer end devices, but as the network size increases, so does the latency due to frequent backoffs. For the highest data rate scenario with the shortest packet transmission time, i.e., SET 3, the latency of LBT is 1.65× higher than broadcast TDMA, and the performance difference is even more significant for SET 1 and 2, with more nodes and a longer transmission time. For instance, to collect data from 9 nodes, LBT requires 1.72× longer latency than broadcast TDMA for SET 1.

##### Unicast TDMA vs. Broadcast TDMA

We now turn to the different modalities of the on-demand TDMA to explore the scalability of the system in terms of latency when pulling data from all the sensing nodes. The RTT latency is plotted in Figure 9b and also reported in Table 3 showing different modes w.r.t. the number of EDs. In the unicast TDMA, the latency linearly increases w.r.t. the number of EDs, independently of LoRa radio settings. This was expected as the sink must transmit a request each time it wants to acquire data from an individual ED and then wait for the data packet, before doing the same with the next node and so on. If the data rate is increased, i.e., SET 2 and 3, unicast mode takes ≈1.65 s and ≈1.3 s, respectively, to collect data from a network size of 9 nodes, whereas broadcast mode provides a dramatic improvement in latency of 3.4× and 4.7×, respectively. This indicates that if the packet transmission time is reduced, the delay performance of broadcast mode is significantly improved, as this causes a proportional decrease in the TDMA frame duration, which is dependent on the time-on-air of the packet. With a lower number of nodes and depending on the settings used, the broadcast mode is capable of collecting data almost every second if necessary.

#### 5.3.3. Energy Efficiency

Next, we study the energy profile of the on-demand TDMA MAC using radio duty-cycle as a metric to indicate the time radios spend in listening or transmitting. In our evaluation, we took into account the radio listening and transmission timings for the LoRa transceiver, as well as the wake-up receiver module. For LPWANs, the most power-hungry process is radio communication; thus, any optimization to extend battery lifetime shall focus on reducing transmission and reception timings, which impacts the overall energy. The radio on times largely depend on the data rate, as well as on the data size. For instance, with a low data rate, the radio can be on for a longer period of time to transmit the same amount of data w.r.t. to the higher data rate. As for most applications, one cannot control the amount of data generated by the sensing devices; thus, selecting the appropriate data rate would provide significant improvements. To this end, we explore the different data rates and their effects on the power using three different configurations.

##### Broadcast TDMA vs. LBT

**Figure 10:**
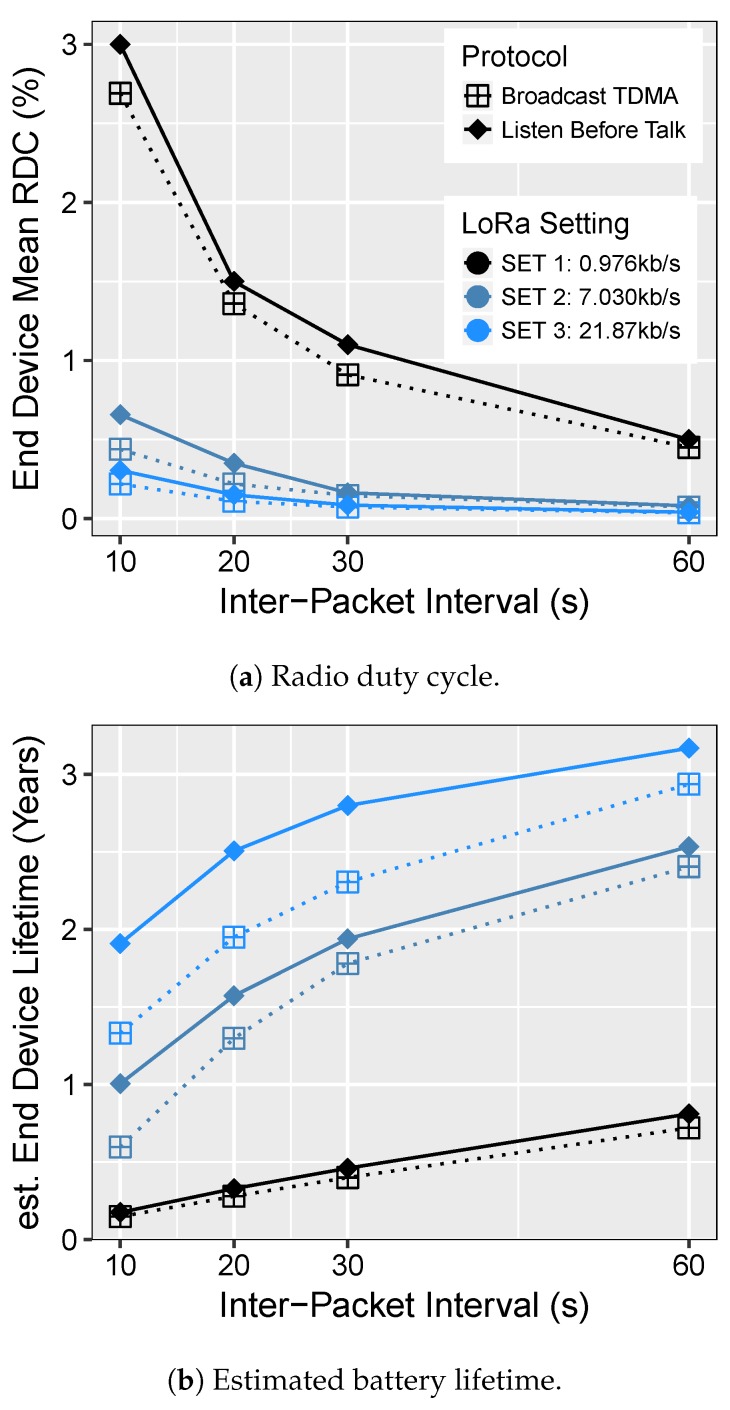
Energy evaluation of on-demand TDMA.

Figure 10a illustrates the mean radio duty cycle ratio of an ED for both protocols. We note that in all scenarios, broadcast TDMA yields lower RDC than LBT. This is attributed to the fact that each node transmits within its specified slot without any extra transmission, allowing nodes to sleep as much as possible. The nodes also do not perform any carrier sensing, thus minimizing idle listening. On the other hand, the nodes employing LBT must check the channel each time before transmission, leading to extra radio activities.

To this end, we see that broadcast TDMA can significantly improve radio listening and transmission time should it allow EDs to use even lower data rates, still abiding by the duty cycle restrictions imposed by LoRaWAN [18]. The radio activity further reduces at a higher data rate with SET 2 and 3. With longer IPIs, the sleep state begins to dominate the system; hence, the RDC of both the protocols start merging as radios spend most of the time in off mode.

Furthermore, we also estimate the end device lifetime to compare the performance of the broadcast TDMA against LBT. On the power consumption side, the addition of the wake-up receiver adds an extra 1.8 μW in continuous listening mode. All these additional costs have been accounted for when calculating the end device lifetime. Device lifetime is a critical metric, as it directly affects the network lifetime. Based on the in-lab power measurements presented in Table 1, the evaluated sensor node draws an average current of 0.56 μA, leading to a theoretical standby time of 244 years on a 1200-mAh lithium polymer battery, only if batteries could last that long. Next, we estimate the end device lifetime when it is actively participating in data collection rounds with varying packet intervals. As seen in Figure 10b, nodes employing the broadcast TDMA scheme can last up to 3 years when polled every minute for data collection. This directly translates into energy savings due to a low radio-on time required by the broadcast TDMA compared to LBT to transmit the same amount of payload (see Figure 10a). Even with shorter trigger intervals, i.e., every 10 s, broadcast TDMA provides a lifetime improvement of up to 1.4× in contrast to LBT. On the other hand, the lifetime of the sensor node is less than a year for the lowest data rate setting w.r.t. both protocols because of the extra power consumed when the radios are active for a long period. Overall, broadcast TDMA demonstrates significant gains in energy that can be achieved over channel sensing schemes for long-range networks.

##### Unicast TDMA vs. Broadcast TDMA

Next, we demonstrate the effectiveness of our proposed MAC by providing a quantitative assessment of the energy consumption to collect data from all the nodes using unicast and broadcast modes. To do this, we conducted testbed experiments where the number of the EDs in the network varied between 1 and 9 with three different LoRa radio settings following the topology shown in Figure 7. In all cases, we report the total energy consumption at the sink to receive the data from all the EDs. The power consumption of the CH together with the EDs is reported in Table 3.

In unicast mode, the energy consumption at the sink and the cluster head linearly increases w.r.t. the number of EDs across all the LoRa settings. This is expected as the sink must send request signals via CH to each ED in a round robin fashion to collect data from all the nodes, spending around 65 mJ per round. A similar trend is also observed at the CH for receiving and relaying the request commands from the sink to the EDs.

In broadcast mode, consumption at the sink is significantly lower for 5 and 9 nodes w.r.t. unicast. This is a combination of the fact that the sink transmits only a single trigger to pull data from all the EDs and the fine-tuning of the idle listening time at the sink. The performance gap is more noticeable when the data rate is high, where the sink and the cluster head consume 5× less energy than unicast trigger to collect data from a network of 9 nodes, i.e., SET 3. The average consumption per ED remains constant per each setting, i.e., 46 mJ for SET 1, as each ED still needs to decode an address embedded in the wake-up beacon for both modes. The extra energy overhead of the protocol is the idle listening at the sink due to the guard time and the continuous listening consumption at the CH. To achieve a data latency in order of a few seconds, this is the trade-off that on-demand TDMA makes by keeping the CH always on for reducing the delay time of the command from the sink to the end devices. In the future, we plan to address this issue by adopting a duty-cycling mechanism at the CH to achieve an energy consumption close to that of end devices.

## 6. Conclusions

This paper presents a new network architecture and an on-demand TDMA MAC protocol leveraging short-range wake-up radios and a LoRa physical layer. On-demand TDMA provides efficient broadcast and unicast service for data transmission and collection, improving the performance of LoRa networks. This work is a stepping stone towards the goal of achieving energy-efficient, yet responsive communication using long-range technology, giving the gateway full control of the network. This is a problem with the LoRaWAN architecture where gateways have minimal or no control over the network and inability to communicate with the end devices upon demand, as discussed in Section 1. The proposed architecture overcomes this drawback by facilitating on-demand triggered communication, where the gateway communicates with the end devices when there are data to be collected. When there is no network activity, the end devices reside in a deep listening state while continuously listening to the wireless channel using an ultra-low power wake-up receiver. The cost of the resulting system, however, slightly increases due to the addition of this extra wake-up receiver module. Nevertheless, on-demand TDMA supports the standard LoRa protocol and can be easily integrated into the LoRaWAN framework for downward data collection.

It has been shown from the testbed experiments that on-demand TDMA significantly improves system scalability and energy efficiency by offering network reliability of 100% with end devices dissipating only 1.83 μW of power during periods of inactivity. We also observed that different LoRa transceiver settings can have significant variations in airtime for a LoRa data packet. Thus, the selection of communication parameters has a tremendous impact on the scalability of a LoRa deployment. While the experimental setup can differ significantly in terms of network architecture, platform design and deployment conditions, our proposed protocol supports a node wake-up delay on the order of milliseconds with a round-trip latency below a second through a two-hop network while sustaining sensing nodes for up to three years.

## Figures and Tables

**Table 1:** Power measurement of the sensor mote in various states at 3.3 V.

**States**	**Power Consumption**
SX1276 in listening mode	50 mW
SX1276 transmitting at +14 dBm (LoRa mode)	250 mW
Mote in deep sleep mode, wake-up receiver listening	1.83 μW
Wake-up receiver (receiving + address decoding)	284 μW
Wake-up transmitter at +14 dBm	260 mW
**Other Parameters**	
Wake-up radio data rate	1 kbps
Wake-up beacon packet size	2 B

**Table 2:** Three different LoRa radio settings (SET) (low, medium and high data rate) used in our experiments.

LoRa Radio Setting	SET 1	SET 2	SET 3
Spreading Factor	12	9	7
Coding Rate	4/6	4/5	4/5
Bandwidth (kHz)	500	500	500
Data Rate (kb/s)	0.976	7.03	21.87
Transmission Power (dBm)	10	10	10
Payload (B)	8	8	8
Preamble Length (symbols)	8	8	8
Carrier Frequency (MHz)	868	868	868
Time-on-air (measured (ms))	264	31	9

**Table 3:** Scalability analysis using different on-demand TDMA operation modes w.r.t. to latency, power and network size.

LoRa Radio Setting		SET 1				SET 2				SET 3			
TDMA Mode	No. of EDs	Sink (mJ)	CH (mJ)	ED (mJ)	RTT Latency (ms)	Sink (mJ)	CH (mJ)	ED (mJ)	RTT Latency (ms)	Sink (mJ)	CH (mJ)	ED (mJ)	RTT Latency (ms)
Unicast	1	65	36.4	46.2	656	12.93	12.83	6.15	183	8	10.63	2.37	139
	5	325	182	231	3280	64.65	64.15	30.75	915	40	53.15	11.85	695
	9	585	327.8	415.8	5904	116.37	115.47	55.35	1647	72	95.67	21.33	1251
Broadcast	1	65	36.4	46.2	656	12.93	12.83	6.15	183	8	10.63	2.37	139
	5	119	90.4	231	1736	20.33	20.23	30.75	331	11.2	13.83	11.85	203
	9	173	144	415.8	2816	27.2	27.6	55.35	479	14.4	17.03	21.33	267

## Abbreviations

The following abbreviations are used in this manuscript:

IoT	Internet of Things
LoRa	long range
TDMA	time division multiple access
MAC	medium access control
WuR	wake-up radio
WuRX	wake-up receiver
WuTX	wake-up transmitter
WuB	wake-up beacon
ISM	Industrial, Scientific and Medical
LPWAN	low power wide area network
CSS	chirp spread spectrum
SF	spreading factor
CR	coding rate
BW	bandwidth
TP	transmission power
OOK	on-off keying
MCU	microcontroller unit
CSMA/CA	carrier sense multiple access with collision avoidance
LBT	listen before talk
CAD	channel activity detection
TI-MAC	transmitter-initiated MAC
RI-MAC	receiver-initiated MAC
PTW	pipelined tone wake-up
W-MAC	wake-up radio MAC
ED	end device
CH	cluster head
ToA	time on air
$G_{t}$	guard time
$N_{id}$	node id
PDR	packet delivery ratio
RDC	radio duty cycle
RTT	round trip time
IPI	inter-packet interval
NB-IoT	Narrow Band Internet of Things

## Appendix A

For a given combination of LoRa setting, spreading factor, coding rate and bandwidth, the total time on air for a given payload size is determined as follows:

(A1) $$ToA_{pkt}=\left[\left(n_{pre}+4.25\right)\frac{1}{R_{sym}}\right]+\left[8+max\left(ceil\left[\frac{8PL-4SF+28+16CRC-20H}{4(SF-2DE)}\right]\left(CR+4\right),0\right)\times \frac{1}{R_{sym}}\right]$$

where:

- $n_{pre}$ is the programmed preamble length
- $R_{sym}$ is the symbol rate
- PL is the payload length in bytes (1–255)
- SF is the LoRa spreading factor (6–12)
- *H* is the packet header, zero when the header is enabled and one when no header is present
- DE is the data rate optimizer, one when enabled, zero otherwise
- CR is the LoRa coding rate [1: 4/5, 2: 4/6, 3: 4/7, 4: 4/8]
